# Nitrogen regulation of the *xyl* genes of *Pseudomonas putida* mt‐2 propagates into a significant effect of nitrate on *m*‐xylene mineralization in soil

**DOI:** 10.1111/1751-7915.12404

**Published:** 2016-08-26

**Authors:** Nanna B. Svenningsen, Mette H. Nicolaisen, Hans Christian B. Hansen, Victor de Lorenzo, Ole Nybroe

**Affiliations:** ^1^ Section for Microbial Ecology and BiotechnologyDepartment of Plant and Environmental SciencesUniversity of Copenhagen1871Frederiksberg CDenmark; ^2^ Section for Environmental Chemistry and PhysicsDepartment of Plant and Environmental SciencesUniversity of Copenhagen1871Frederiksberg CDenmark; ^3^ Systems and Synthetic Biology ProgramCentro Nacional de Biotecnología (CNB‐CSIC)Madrid28049Spain

## Abstract

The nitrogen species available in the growth medium are key factors determining expression of *xyl* genes for biodegradation of aromatic compounds by *Pseudomonas putida*. Nitrogen compounds are frequently amended to promote degradation at polluted sites, but it remains unknown how regulation observed in the test tube is propagated into actual catabolism of, e.g. *m*‐xylene in soil, the natural habitat of this bacterium. To address this issue, we have developed a test‐tube‐to‐soil model system that exposes the end‐effects of remediation practices influencing gene expression of *P. putida* mt‐2. We found that NO_3_^−^ compared with NH_4_^+^ had a stimulating effect on *xyl* gene expression in pure culture as well as in soil, and that this stimulation was translated into increased *m*‐xylene mineralization in soil. Furthermore, expression analysis of the nitrogen‐regulated genes *amtB* and *gdhA* allowed us to monitor nitrogen sensing status in both experimental systems. Hence, for nitrogen sources, regulatory patterns that emerge in soil reflect those observed in liquid cultures. The current study shows how distinct regulatory traits can lead to discrete environmental consequences; and it underpins that attempts to improve bioremediation by nitrogen amendment should integrate knowledge on their effects on growth and on catabolic gene regulation under natural conditions.

## Introduction

Approaches used in environmental biotechnology to bring about pollutant degradation, i.e. bioremediation, rely on the activities of catabolic microorganisms in complex environments. In their natural habitat, these microorganisms encounter numerous exogenous factors that can affect their growth and general metabolic activity, and even their ability to express specific genes involved in pollutant degradation (de Lorenzo, 2008). Hence, the impact of environmental factors on catabolic microorganisms is of paramount significance for the optimal exploitation of specific degrader microorganisms in bioaugmentation, where microorganisms selected for catabolic performance under laboratory conditions are brought back into their natural habitat.

However, how these properties are regulated by microorganism in the environment is at present not clear (Meckenstock *et al*., 2015).

**Figure 1:**
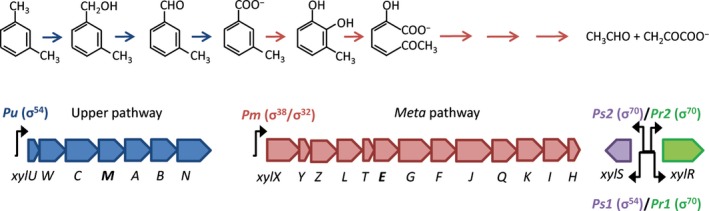
Degradation of *m*‐xylene and organization of the *xyl* structural and regulatory genes of the TOL pathway on pWW0. Enzymes of the upper pathway catalyse the sequential oxidation of one methyl group of *m*‐xylene resulting in 3‐methyl benzoate (3MB), which is further converted to tricarboxylic acid cycle intermediates by enzymes encoded in the *meta* pathway operon. Six promoters are involved in the expression of the two catabolic gene clusters. Transcription of the upper pathway is initiated from the σ^54^‐dependent promoter *Pu*, while the meta pathway is transcribed from *Pm* that requires either σ^32^ (σ^H^) or σ^38^ (σ^S^). Furthermore, two regulatory proteins, XylR and XylS, are involved in the tightly controlled expression of the entire TOL pathway that additionally requires the presence of specific effector molecules, *m*‐xylene and 3MB, and the chromosomal‐encoded HU and IHF proteins. The master regulator XylR, encoded by the *xylR* gene, is transcribed from two σ^70^‐dependent tandem promoters, and is involved in activation of *Pu* and the σ^54^‐dependent *Ps1* promoter of *xylS*, encoding the meta pathway regulator, XylS. In addition, *xylS* is constitutively expressed from the σ^70^‐dependent promoter *Ps2*. The two catabolic genes employed as proxies of TOL pathway expression in this study, *xylM* and *xylE* from the upper and *meta* pathway, respectively, are shown in bold.

*Pseudomonas putida* mt‐2, which carries the catabolic TOL plasmid, pWW0, enabling *m*‐xylene and toluene degradation is a safe and well‐studied paradigm organism for applications in bioaugmentation (de Lorenzo *et al*., 2013). The catabolic *xyl* genes on pWW0, which are involved in *m*‐xylene and toluene degradation, are organized in the upper and lower/*meta* TOL pathway operons (Fig. 1). The significance of environmental factors for *xyl* gene expression has been intensively studied in pure culture model systems (Velázquez *et al*., 2006; Del Castillo and Ramos, 2007). Some of these studies have revealed that nitrogen sources, e.g. NH_4_^+^ and NO_3_^‐^, influence *xyl* gene expression in mt‐2 (Velázquez *et al*., 2006; Huang *et al*., 2008). Expression of *xyl* genes of the upper TOL pathway is regulated by the sigma factor, σ^54^, which was initially identified as a nitrogen specific sigma factor (Ramos *et al*., 1997; Cases *et al*., 2003). Hence, this global regulator might modulate substrate‐specific induction of the TOL pathway in response to altered environmental conditions caused by the nitrogen supplements (Commichau *et al*., 2006; Hervás *et al*., 2009).

Nitrogen is an important limiting element for bacterial growth in many environments, in particular at hydrocarbon contaminated sites (van Veen *et al*., 1997; Jensen and Nybroe, 1999; Walecka‐Hutchison and Walworth, 2006), and several studies on bioremediation of hydrocarbon contaminated soils and sediments have shown the significance of inorganic nitrogen amendments for *in situ* pollutant biodegradation (Lindstrom *et al*., 1991; Davis and Madsen, 1996; Aislabie *et al*., 2012). The chosen source of nitrogen to add in bioremediation studies is NH_4_^+^ that can be taken up and assimilated directly by the bacterial degraders (Walecka‐Hutchison and Walworth, 2006; Komilis *et al*., 2010). Hence, it becomes of interest whether the different effects of NH_4_^+^ and NO_3_^−^ on catabolic gene expression by *P. putida* mt‐2, which have been revealed by well‐controlled pure culture conditions, where typically one parameter was changed at a time (Velázquez *et al*., 2006; Hervás *et al*., 2008) manifest also in a complex environmental context (de Lorenzo, 2008). An additional, unsettled issue is how tightly changes in *xyl* gene expression are coupled to the functional outcome of the pathway, i.e. changes in *m*‐xylene biodegradation in a soil system.

The objective of current study was to address whether the test tube effects of nitrogen on catabolic gene expression by *P. putida* mt‐2 propagate into an actual environmental scenario. To this end we established an experimental model system allowing us to compare the effect of specific nitrogen sources (NH_4_^+^ vs. NO_3_^−^) on the transcriptional dynamics of *P. putida* mt‐2 catabolic *xyl* genes in pure culture and after introduction to natural soil with different nitrogen regimes. In either systems, we focused on expression of *xylM* and *xylE* of the upper and lower TOL pathway, respectively, as the enzyme products of these two genes are regarded as key enzymes in the biodegradation of toluene and *m*‐xylene (Hendrickx *et al*., 2006; Martínez‐Lavanchy *et al*., 2010). In addition, sensing of the nitrogen status by *P. putida* mt‐2 cells was compared for pure culture and soil systems through expression of the *amtB* and *gdhA* genes signalling NH_4_^+^ deficiency and surplus respectively (Hervás *et al*., 2008). The system further allowed us to examine whether changes in gene expression in soil were translated into actual variations in biodegradation performance.

## Results

### Nitrogen source impact on expression of *xylM* and *xylE* in pure culture

**Figure 2:**
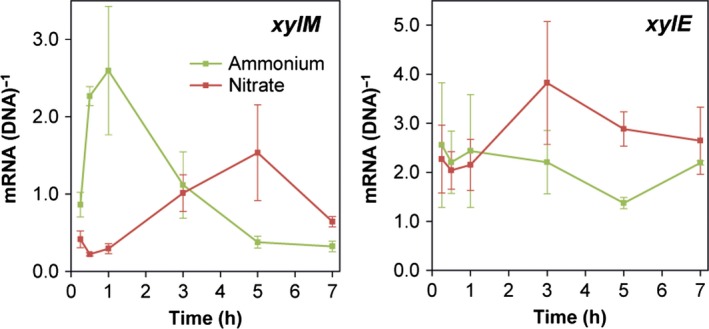
Transcriptional dynamics of *xylM* and *xylE* by *P. putida* mt‐2 incubated in the presence of *m*‐xylene vapours in M9 medium with either 10 mM NH_4_^+^ (green) or 10 mM NO_3_^−^ (red) as sole N‐source. Samples were withdrawn between 15 min and 7 h after the shift in N‐source. Data are mean values of mRNA normalized to DNA copies of the corresponding genes from triplicate cultures, and error bars represent standard error of mean.

**Figure 3:**
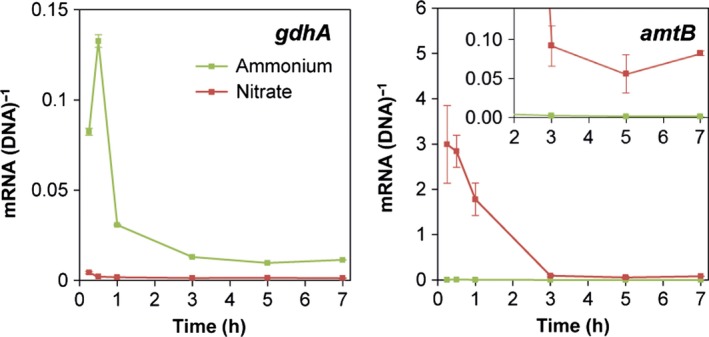
Transcriptional dynamics of the N‐regulated genes *gdhA* and *amtB* by *P. putida* mt‐2 incubated in the presence of *m*‐xylene vapours in M9 medium with either 10 mM NH_4_^+^ (green) or 10 mM NO_3_^−^ (red) as sole N‐source. Insert in the right panel shows expression of *amtB* after 2–7 h on the same scale as expression of *gdhA*. Data are mean values of mRNA normalized to DNA copies of the corresponding genes from triplicate cultures, and error bars represent standard error of mean.

Initially, we performed a detailed analysis of the temporal dynamics of *xylM* and *xylE* expression for exponentially growing *P. putida* mt‐2 incubated with *m*‐xylene and either NH_4_^+^ or NO_3_^−^ as nitrogen source. NH_4_^+^ had an initial stimulating effect on *xylM* expression compared with NO_3_^−^ (*P* = 0.006) within the first hour after shifting the nitrogen source (Fig. 2). However after 3–5 h, the expression pattern shifted, and while *xylM* expression in NH_4_^+^ medium decreased, a delayed expression peak in NO_3_^−^ medium was observed. Hence after 5 h, expression of *xylM* in NO_3_^−^ medium was significantly higher than expression in NH_4_^+^ medium (*P* = 0.027). In contrast, we observed no difference in *xylE* expression between the two treatments until after 3 h (Fig. 2). At this time, NO_3_^−^ stimulated *xylE* expression compared with NH_4_^+^, and after 5 h the difference to expression in NH_4_^+^ medium was statistically significant (*P* = 0.015). Cultures in NH_4_^+^ medium as well as cultures in NO_3_^−^ medium reached stationary phase after 1–3 h according to qPCR quantification of *xylM* and *xylE* copy numbers (data not shown). Hence, the differences in expression levels were not related to differences in growth phase between cultures,

Expression of the nitrogen‐regulated genes *amtB* and *gdhA*, encoding a high‐affinity NH_4_^+^ transporter expressed during NH_4_^+^ limitation, and glutamate dehydrogenase, reported to be highly expressed in the presence of NH_4_^+^, respectively (Hervás *et al*., 2008), was measured to determine whether the two nitrogen sources were perceived differentially by the cells (Fig. 3). In cultures incubated with NO_3_^−^, *amtB* expression was initially more than 100‐fold higher than in cultures incubated with NH_4_^+^ as nitrogen source. The *amtB* expression then declined but remained significantly higher than for cells incubated in NH_4_^+^ medium for at least 7 h (Fig. 3, insert). In contrast, *gdhA* expression initially peaked for cells shifted from a spent to a fresh NH_4_^+^ medium, but remained low for cells grown with NO_3_^−^. The expression of *gdhA* hereafter decreased to a low and steady level that remained highest (*P* < 0.05) for cells cultured with NH_4_^+^ throughout the entire sampling period.

In conclusion, the nitrogen sources impact *xyl* gene expression in this pure culture system with NO_3_^−^ having a delayed, but overall stimulating effect compared with NH_4_^+^. Furthermore, the expression of *amtB* and *gdhA* provided information on the NH_4_^+^ availability to *P. putida* mt‐2, and we consequently transferred this monitoring system for use in subsequent soil experiments.

### Response of *P. putida* mt‐2 to changing nitrogen‐conditions in soil microcosm

A soil microcosm was established in which nitrogen limitation was brought about by pre‐incubation with ground barley straw (Jensen and Nybroe, 1999) so that the influence of added nitrogen sources could be addressed with minimal interference from indigenous nitrogen pools. After incubation with straw for 7 days, the soil contained ~0.1 mmol kg^−1^ soil of water soluble NO_3_^−^ and NH_4_^+^ as determined by chemical analyses. This soil is referred to as N‐limited soil hereafter. *P. putida* mt‐2 was then introduced to the N‐limited soil and exposed to *m*‐xylene. Nitrogen in the form of NaNO_3_ or NH_4_Cl was added to reach a concentration of 10 mmol kg^−1^ soil respectively. The pH of the NH_4_^+^‐amended soil (pH 5.8) decreased slightly to below the pH of the NO_3_^−^‐amended soil (pH 6.5) at the end of the 46‐h incubation period.

**Figure 4:**
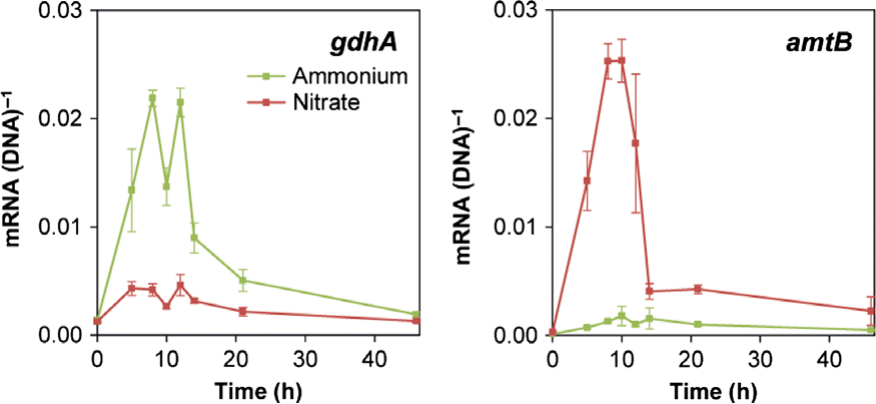
Dynamics of expression of the N‐regulated genes *gdhA* and *amtB* by *P. putida* mt‐2 inoculated into N‐limited soil amended with 10 mmol kg^−1^ soil NH_4_^+^ or NO_3_^−^. Data are mean values of mRNA normalized to DNA copies of the corresponding genes from triplicate soil setups, and error bars represent standard error of mean.

Addition of NO_3_^−^ to the N‐limited soil gave rise to an increase in expression of *amtB* compared with that seen for the N‐limited soil supplemented with NH_4_^+^ (Fig. 4). Expression peaked at 8–10 h incubation, i.e. later than in liquid culture (Fig. 3), and after 12 h, *amtB* expression was again downregulated; nonetheless, the expression lasted longer than in liquid culture. In contrast, *gdhA* expression was strongly upregulated in the N‐limited soil receiving NH_4_^+^, but only increased slightly from the background level in NO_3_^−^‐amended soil (Fig. 4). Downregulation of *gdhA* in NH_4_^+^‐amended soil also appeared, but the downregulation occurred slightly later than the downregulation of *amtB* in NO_3_^−^‐amended soil. The levels of *amtB* and *gdhA* expression were comparable, which contrast the ~20‐fold higher expression of *amtB* than *gdhA* in pure culture (compare Figs 3 and 4).

In conclusion, sources of inorganic nitrogen in the soil directly affect expression of the selected indicator genes involved in nitrogen uptake and transformations. Although the expression patterns were not identical to those recorded for *P. putida* mt‐2 in liquid culture, they reveal that soil amended with NO_3_^−^, unlike soil amended with NH_4_^+^, is perceived as being NH_4_^+^ deficient by the introduced cells.

### Nitrogen source impact on expression of *xylM* and *xylE* and on *m*‐xylene mineralization in soil

**Figure 5:**
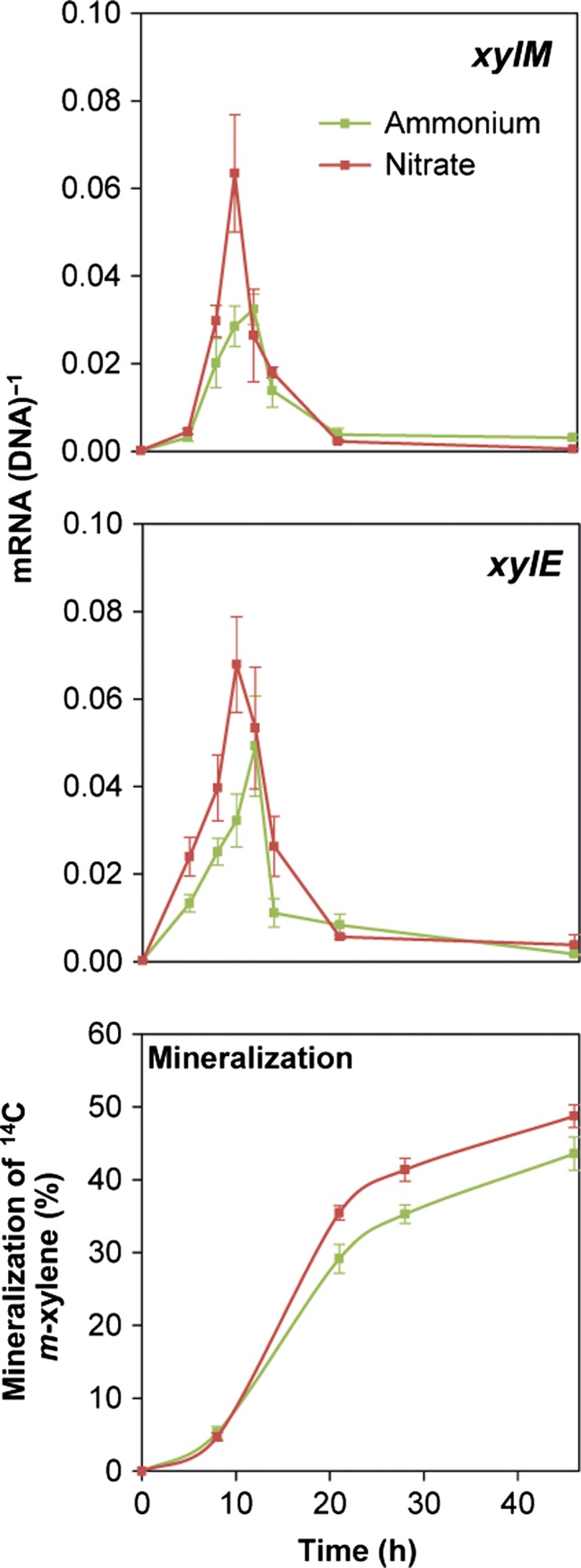
Transcriptional dynamics of *xylM* and *xylE* (two upper panels), and mineralization of *m*‐xylene (lower panel) in N‐limited soil amended with 10 mmol kg^−1^ soil NH_4_^+^ or NO_3_^−^. Gene expression data are mean values of mRNA normalized to DNA copies of the corresponding genes from triplicate soil set‐ups, and error bars represent standard error of mean. Mineralization data are mean values from triplicate soils and error bars represent standard error of mean.

In soils receiving NO_3_^−^, expression of *xylM* and *xylE* peaked after 10 h of incubation, while the expression peaks in NH_4_^+^‐amended soils appeared slightly delayed after 12 h (Fig. 5). The NO_3_^−^ amendment resulted in higher expression of both *xyl* genes in the ascending part of the expression curves. For *xylM* the expression was significantly (*P* = 0.044) higher (approximately twofold) in NO_3_^−^‐amended soil when comparing the peak after 10 h with the peak after 12 h in the NH_4_^+^‐amended counterpart. For *xylE*, there was nevertheless only a tendency towards a higher expression peak in the NO_3_^−^ amended soil (*P* = 0.30).

Compared with observations made for pure cultures, the initial stimulatory effect of NH_4_^+^ on *xylM* expression was not seen in soil, and there was a delay in the stimulatory effect on *xylM* and *xylE* expression exerted by NO_3_^−^ (compare Figs 3 and 5). Importantly, the higher *xyl* gene expression in NO_3_^−^‐amended soils was reflected in a significantly (*P* = 0.047) higher mineralization of *m*‐xylene after 21 h and onwards when compared with soils amended with NH_4_^+^ (Fig. 5). In N‐limited soil that did not receive NO_3_^−^ or NH_4_^+^, the mineralization did not differ from mineralization in NH_4_^+^‐amended microcosms (data not shown).

In the current experiment, *P. putida* mt‐2 was introduced to a natural soil that may contain indigenous xylene mineralizing populations carrying *xyl* genes. To determine the extent to which these populations contributed to our expression and mineralization analyses, we showed that the soil contained less than 10^3^ copies g^−1^ soil of *xyl* genes. Finally, mineralization analysis in soil microcosm without inoculated mt‐2 cells revealed that mineralization by indigenous *m*‐xylene‐degrading soil bacteria accounted for a minor part (~10%) of ^14^C‐CO_2_ build up (data not shown). Consequently, *m*‐xylene mineralization could be ascribed to the inoculated *P. putida* mt‐2 cells.

In conclusion, *xyl* gene expression by *P. putida* mt‐2 in soil was stimulated more by NO_3_^−^ than by NH_4_^+^. The same was observed for liquid cultures, however with a different temporal expression pattern. Importantly, the changes in *xyl* gene expression in response to the different nitrogen sources served as descriptors of corresponding changes in *m*‐xylene mineralization in the soil system.

## Discussion

Environmental conditions affecting *xyl* gene expression by *P. putida* mt‐2 introduced to soil has to the best of our knowledge not previously been clarified. In complex soil environments, introduced degrader bacteria are exposed to a variety of biotic and abiotic stress factors, which might not resemble situations tested individually under standard liquid culture conditions. At the current time, it remains obscure how bacterial metabolism is regulated in their natural habitat (Meckenstock *et al*., 2015), and whether regulatory concepts derived from liquid culture studies (Duetz *et al*., 1996; Commichau *et al*., 2006; Hervás *et al*., 2008; Pflüger‐Grau and Görke, 2010) are valid for catabolic bacteria exposed to the plethora of challenging conditions in their natural environment. Hence, the current study integrates experiments in a pure culture system and a soil model system. Importantly, our analyses couples changes in *xylM* and *xylE* gene expression to changes in the output of the catabolic pathway in question, *m*‐xylene biodegradation, as transcript abundance and their cognate processes is often not correlated under environmental conditions (Rocca *et al*., 2014).

Our results on *xyl* gene regulation by nitrogen sources in pure culture expand the results of Velázquez and colleagues (Velázquez *et al*., 2006), who determined *xyl* gene expression by microarray technology and by Huang and coworkers (Huang *et al*., 2008), who detected increased *Pu* promoter activity with NO_3_^−^ as nitrogen source at single time points. Interestingly, the stimulation of *xyl* gene expression by NO_3_^−^ observed in the current study occurred later than in previous studies emphasizing the importance of monitoring temporal dynamics of gene expression.

NH_4_^+^ is considered the preferred nitrogen source for bacteria, as its assimilation is less energy‐expensive as compared with assimilation of NO_3_^−^ that first needs conversion into NH_4_^+^ before it is assimilated via glutamine syntethase–glutamate synthase (Magasanik, 1993; Merrick and Edwards, 1995; Leigh and Dodsworth, 2007). The physiological change following substitution of NH_4_^+^ with NO_3_^−^ might consequently be due to introduction of a poor nitrogen source, although Velázquez *et al*. (2006) only noted a weak induction of indicator genes for nitrogen starvation stress in *P. putida* mt‐2 exposed to NO_3_^−^. To monitor the availability of NH_4_^+^ to mt‐2 cells, we quantified the expression of two genes, *amtB* and *gdhA* that are under control of the major nitrogen‐associated transcriptional regulator, NtrC. The *amtB* gene encodes a high‐affinity NH_4_^+^ transporter. It belongs to the NH_4_^+^ transport family of proteins ubiquitous to all bacteria, and is blocked under conditions of nitrogen excess (Coutts *et al*., 2002; Javelle and Merrick, 2005; Leigh and Dodsworth, 2007). Expression of *amtB* in *P. putida* is stimulated under nitrogen limiting conditions established, e.g. during growth on serine (Hervás *et al*., 2008; Yeom *et al*., 2010). The glutamate dehydrogenase gene, *gdhA*, on the other hand is reported to be expressed under conditions of normal nitrogen access and is actively repressed by nitrogen limitation in *P. putida* (Hervás *et al*., 2010). Our observation of high *amtB* and very low *gdhA* expression in NO_3_^−^‐amended cultures over the entire time‐course examined here indicates that mt‐2 sensed this medium as being NH_4_^+^ deficient. The opposite expression pattern recorded during growth with NH_4_^+^ demonstrates that, in concert, *amtB* and *gdhA* function as indicators for the cellular nitrogen status. Interestingly, the expression of *amtB* and *gdhA* is highly dynamic within 1–3 h where after expression is downregulated to a steady level likely because the nitrogen fluxes have reached balance. Peaks in expression have previously been observed, e.g. for the *Cupriavidus pinatubonensis tfdA* gene involved in herbicide catabolism just after exposure to the substrate, and for the *P. putida* catalase gene *katA* just after exposure to hydrogen peroxide (Svenningsen *et al*., 2015 and unpublished observations). We speculate that a pool of enzymes is produced during the burst in gene expression that is able to carry out the requested function for an extended time period. Again, our data underline that data obtained from sampling at single timepoints in gene expression studies might lead to incorrect conclusions.

To be able to assess the nitrogen source impact on *m*‐xylene biodegradation in soil, we reduced the readily accessible soil nitrogen pool through incubation with barley straw. Due to a C:N ratio higher than the average bacterial C:N ratio, this treatment immobilizes nitrogen in non‐sterile soil (Geisseler *et al*., 2010). The expression of the two nitrogen‐regulated genes *amtB* and *gdhA* showed initial peaks as discussed above for liquid cultures. However, the response was slower and we also noted subtle differences in induction levels between pure cultures and soil that could be explained by the NH_4_^+^ levels in the two systems. Hence, the weaker induction of *amtB* in NO_3_^−^‐amended soil than in liquid NO_3_^−^‐amended medium as well as the weak induction of *gdhA* in NO_3_^−^‐amended soil suggest that small amounts of an easily available nitrogen source were available in the soil. Indeed, chemical analysis showed that the nitrogen‐limited soil still contained 0.1 mmol kg^−1^ of water soluble NH_4_^+^ after the straw pre‐treatment. This NH_4_^+^ pool has been available even to the cells introduced to NO_3_^−^‐amended soils. Hence, our results indicate that mt‐2 cells are able to sense and respond to indigenous nitrogen pools in the soil, and they demonstrate that the *amtB* and *gdhA* genes are valid indicator genes for studying the bioavailability of NO_3_^−^ versus NH_4_^+^ to *P. putida* mt‐2 in natural soil.

The higher expression of *xyl* genes in response to *m*‐xylene in NO_3_^−^‐amended than NH_4_^+^‐amended soil was in general agreement with observations made for pure cultures. Obviously, *xyl* gene induction in the soil was slower than in liquid culture. This might be caused by sorption of *m*‐xylene to soil organic matter. However, the slower response was even recorded for expression of genes involved in nitrogen metabolism as discussed above. Hence, the delay might be ascribed to the downshift that the cells experience upon transfer to the oligotrophic soil environment (van Veen *et al*., 1997; Koch *et al*., 2001) with a high complexity of potential stressors (including the *m*‐xylene carrier hexane) that the cells need to deal with (Daurabas and Chakrabarty, 1992; Velázquez *et al*., 2006). Importantly, the robustness of our gene expression system permitted us to relate small differences in gene expression in soil under the two nitrogen regimes to a significant difference in total mineralization of the added *m*‐xylene. This is important because discrepancy between the amounts of transcripts and their corresponding protein abundance and functional activity is occasionally observed (Poblete‐Castro *et al*., 2012). Regulation of gene expression is the first and most direct cellular response to changed environmental conditions in prokaryotes. We indeed observed that *xyl* gene induction preceded *m*‐xylene mineralization in soil, probably reflecting the time required for establishing a pool of catabolic enzymes in the cells. Comparable time‐courses have been observed for *tfdA* gene expression and MCPA herbicide mineralization by *C. pinatubonensis* introduced to soil (Nicolaisen *et al*., 2008). When combined with ^14^C‐mineralization assays, we suggest that *xyl* transcript analysis provides robust insight into factors controlling *m*‐xylene biodegradation, but for future studies proteome analyses of the catabolic enzymes could be of considerable interest.

It remains to be clarified exactly how nitrogen status affect *xyl* gene expression. The upper TOL pathway promoter *Pu* as well as the *Ps1* promoter of the *meta* pathway transcriptional regulator XylS are controlled by the sigma factor, σ^54^, encoded by the *rpoN* gene (Ramos *et al*., 1997; Cases *et al*., 2003; Shingler, 2003) (Fig. 1). Velázquez and coworkers (Velázquez *et al*., 2006) proposed that NO_3_^−^ could increase *xyl* gene expression by a mechanism involving stimulation of the two σ^54^ dependent TOL pathway promoters by increasing the amount of σ^54^‐bound core RNA polymerases; a condition that might occur during assimilation of NO_3_^−^‐derived NH_4_^+^ through glutamine syntethase–glutamate synthase (Moreno‐Vivián *et al*., 1999; Velázquez *et al*., 2006). An alternative mechanism suggested by Aranda‐Olmedo *et al*. (2005) is that the nitrogen phosphotransferase system, PTS^Ntr^, involved in nitrogen metabolism (Pflüger‐Grau and Görke, 2010), also interferes with activation of the σ^54^‐dependent TOL pathway promoters via their effector molecules; hence, this mechanism is more related to the interplay between nitrogen starvation/metabolism and carbon metabolism. The observation that xylene mineralization was comparable in natural soil and nitrogen‐depleted soil amended with NH_4_^+^ might suggest that the higher mineralization in NO_3_^−^‐amended soils is a direct response to NO_3_^−^. However, to the best of our knowledge, a NO_3_^−^‐sensing mechanism has not been described for *P. putida*.

The current methodological approach allowed us to gain insight into regulation of catabolic gene expression of *P. putida* mt‐2 by environmental factors under close‐to‐natural soil conditions. Although temperature and water content were kept constant, the mt‐2 cells introduced to a natural soil would be confronted with some spatial heterogeneity, with possible competition or collaboration from indigenous microorganisms, and with realistic indigenous pools of carbon sources and nutrients. Our study underscores that global regulation of catabolic genes acts beyond direct substrate induction. Furthermore, regulatory patterns emerge in our soil model systems that are comparable to those observed in liquid cultures. Nevertheless, we even observe noteworthy differences in terms of temporal dynamics and induction levels. Interestingly, we have seen that environmental regulation of *xyl* genes in pure culture does not correspond to regulation in soil, when changing the available carbon sources (NB Svenningsen, unpublished results). Hence, more effort could be devoted to deciphering the environmental factors that affect expression of these genes in the soil.

With the current model system in hand, we have a good basis for investigating the ‘behaviour’ of *P. putida* mt‐2 under realistic conditions in different soils and considering other stressors that could influence the potential for biodegradation. From both a basic and an applied perspective, it is a key issue to understand the *in situ* environmental conditions able to stimulate the biodegradative potential of a particular organism (Cases and de Lorenzo, 2005). Although the specific nitrogen sources available for inoculated degrader bacteria will influence their growth potential, and therefore the potential for pollutant degradation on the long run, our data show that specific nitrogen sources in soil also affect the expression of catabolic genes of degrader bacteria. Hence, we suggest that attempts to improve bioremediation of pollutants from contaminated sites should integrate knowledge on environmental effects on growth as well as on catabolic gene regulation under natural conditions.

## Experimental procedures

### Bacterial strain and growth conditions

*Pseudomonas putida* mt‐2 harbouring the TOL plasmid, pWW0 (Greated *et al*., 2002) that enables it to degrade among others toluene, *m*‐ and *p*‐xylene, was obtained from Deutsche Sammlung von Mikroorganismen und Zellkulturen GmbH, Braunschweig, Germany (DSM‐6125). For all experiments, the strain was pre‐cultured over night at 28°C with agitation at 150 r.p.m. in M9 minimal medium (6.0 g L^−1^ Na_2_HPO_4_, 3.0 g L^−1^ KH_2_PO_4_, 0.5 g L^−1^ NaCl, 1.0 g L^−1^ NH_4_Cl, 0.25 g L^−1^ MgSO_4_·7 H_2_O, 0.015 g L^−1^ CaCl_2_·2 H_2_O) supplemented with 5 mM Na‐succinate. For pure culture experiments, the overnight cultures were diluted in fresh M9 medium as specified in following section. For soil experiments, cultures were harvested by centrifugation (5000 *g*, 5 min, 21°C), and cells were subsequently washed twice and re‐suspended in 1× phosphate‐buffered saline (PBS). Cell densities were calculated based on measurements of optical density at 600 nm with OD = 1 corresponding to 10^9^ cells ml^−1^ measured by standard CFU counting on LB agar.

### Liquid culture experiment and sampling for nucleic acid extraction

Overnight cultures of *P. putida* mt‐2 in M9 medium (OD_600nm_ ~0.8) were diluted 100 times in fresh medium and exposed to vapours of *m*‐xylene (Sigma‐Aldrich, St. Louis, MO, USA) stemming from a 1:5 dilution in dibutyl phthalate (Sigma‐Aldrich) in sealed flasks, basically as described in Velázquez *et al*. (2006). At OD_600nm_ ~0.5, cells were washed twice in PBS before resuspending them in N‐free M9 medium supplemented with either 10 mM NO_3_^−^ or NH_4_^+^ as nitrogen source and incubated in the presence of *m*‐xylene at 28°C with agitation at 150 r.p.m. The bottles were sealed and kept closed during the incubation. At 15 min, 30 min, 1, 3, 5 and 7 h after the shift in nitrogen sources, samples were withdrawn with a sterile syringe through a septum in the cap. Cells were pelleted by centrifugation at 4°C (10,000 *g*, 2 min), frozen in liquid N_2_ and immediately stored at −70°C until nucleic acid extraction.

### Soil characteristics and soil model set‐up

Agricultural soil was collected from the Ap horizon (0–34 cm) of a soil profile located 20 km west of Copenhagen at 55°40′ N and 12°18′ E on the experimental farm Rørrendegård of Copenhagen University. The soil was stored in closed containers at 4°C until use. A subsample of soil was used for soil characterization. The soil pH was 6.8, determined in a 1:1 soil–water suspension. The particle size distribution was 22% clay, 13% silt, 29% course sand and 36% fine sand, and was determined by sieving and sedimentation. Determination of total soil carbon was carried out by dry combustion, and total nitrogen was determined by Kjeldahl digestion. The total carbon content was 1.1% (w/w) and total nitrogen was 0.13% (w/w).

Prior to the experiment, the moist soil was passed through a 2 mm mesh sieve, and a subsample of soil was heated at 105°C for 24 h to determine the water content. To obtain depletion of nitrogen, ground barley straw (2.5% w/w) was mixed into the soil, which was subsequently incubated at 20°C for 1 week in order to immobilize soil nitrogen (Jensen and Nybroe, 1999). Straw residues were then removed by passing the soil through the 2 mm mesh sieve again. Next the moisture content was adjusted to 18% of soil dry weight with sterile filtered demineralized water (MilliQ; Merck Millipore, Darmstadt, Germany), taking into account the subsequent addition of cells and nitrogen solutions. Washed stationary phase *P. putida* mt‐2 cells were inoculated into the soil to reach a cell density of approximately 10^8^ cells g^−1^ soil. Cells were mixed into the soil by hand in a diffusion limited soil sampling bag (Rilsan; Rotek A/S, Sønder Felding, Denmark) together with NaNO_3_ or NH_4_Cl solutions. Nitrogen solutions were added to obtain concentrations of 10 mmol N kg^−1^ soil. A mixture of *m*‐xylene and ^14^C‐labelled *m*‐xylene (8000 dpm g^−1^ soil) in hexane as carrier was then mixed into the soil to a concentration of *m*‐xylene of 200 mg kg^−1^. Subsequently, 20 g soil was distributed in triplicate into glass bottles, and glass vials containing 1 M NaOH were placed on top of the soil. From each bottle, triplicate soil samples were immediately taken, in which radioactivity was measured by liquid scintillation counting to be able to correct for loss of *m*‐xylene evaporating during the set‐up. Bottles were sealed with screw caps and incubated at 20°C in the dark.

Mineralization of *m*‐xylene was measured during the incubation period by liquid scintillation counting of collected ^14^C‐CO_2_ in the NaOH using a Beckman LS1801 scintillation counter (Beckman Coulter, Copenhagen, Denmark) after mixing the 1 ml NaOH samples with 4 ml scintillation cocktail (Optiphase ‘Hisafe'3; Perkin Elmer, Skovlunde, Denmark) followed by incubation in the dark for 2 h. After 0, 5, 8, 10, 12, 14, 21 and 46 h of incubation, soil samples of 0.5 g were obtained. For each sampling, the screw caps were shortly removed from the bottles. Soil samples were immediately frozen in liquid N_2_ and stored at −70°C until nucleic acid extraction.

In addition to the treatments described above, a control soil microcosm without inoculation of mt‐2 cells, plus addition of *m*‐xylene was prepared to test for background mineralization of *m*‐xylene and direct capture of ^14^C *m*‐xylene in NaOH traps. Furthermore, a control with inoculation of mt‐2 cells but without addition of *m*‐xylene was set up to test for if the soil itself stimulates induction of the *xyl* genes in soil.

Additionally, at the beginning and in the end of incubation, soil water samples were collected for determination of pH and concentrations of NH_4_^+^ and NO_3_^−^ associated to soil water (i.e. the directly bioavailable part). This was done by vortexing 0.15 g soil with 1.5 ml MilliQ water, followed by 1 h of shaking at 200 r.p.m. Finally, samples were centrifuged (10,000 *g*, 5 min) and analyses of pH and concentrations of NO_3_^−^ and NH_4_^+^ were conducted on the supernatants.

### Nucleic acid extraction and quantitative PCR

For extraction of nucleic acids from pure culture samples, the AllPrep DNA/RNA Mini Kit (Qiagen, Manchester, UK) was used according to the manufacturer's protocol with addition of a lysozyme pre‐treatment step at room temperature for 20 min (100 μl of 1 mg ml^−1^ in 10 mM Tris‐Cl buffer, pH 8, per sample of 100 μl) as the only modification. From soil samples, DNA and RNA were co‐extracted by the phenol–chloroform method as formerly described (Nicolaisen *et al*., 2008). Subsequent to extraction DNA was eliminated from RNA samples by treating aliquots of each nucleic acid sample with RQ1 RNase‐free DNase 1 (Promega, Nacka, Sweden) according to the manufacturer's protocol. cDNA was synthesized immediately thereafter by using 2 μl subsamples of each DNase‐treated extract as template for reverse transcription (RT) with the Omniscript RT Kit (Qiagen). DNase‐treated control reactions were prepared in parallel for RNA samples without addition of the reverse transcriptase (RT) to ensure the absence of DNA contamination. RT reactions were prepared with 400 ng of random hexamer primers (Promega), 4 U of SUPER RNase inhibitor (Ambion, Austin, TX, USA) and reagents provided in the kit for a final volume of 20 μl. Incubation conditions were followed as recommended by the manufacturer. Resulting cDNA samples were stored at −20°C until use in qPCR.

Previously published primers were used to quantify the expression of *xylM*,* xylE* (Martínez‐Lavanchy *et al*., 2010), *amtB* and *gdhA* (Hervás *et al*., 2008). qPCR reactions were prepared in 20 μl with 10 μl Brilliant II SYBR Green QPCR Master mix (Stratagene, La Jolla, CA, USA), 0.3 μM of each primer and 1 mg ml^−1^ BSA. Thermal cycling conditions were following: an initial cycle of 95°C for 10 min, followed by 40 cycles of 95°C for 30 s, primer annealing at the temperatures stated in (Hervás *et al*., 2008; Martínez‐Lavanchy *et al*., 2010) for 45 s and an elongation step at 72°C for 1 min. Subsequently a melting curve was run. All cDNA and DNA samples were diluted 1:10 before the qPCR. To check for possible contamination of RNA samples by genomic DNA, diluted samples of RNA were analysed by qPCR as well. Ct values for pure culture samples were related to a standard curve prepared from 10‐fold dilutions of DNA extracted from 1 ml of liquid culture with OD_600nm_ of 0.8. For soil samples, a standard curve was prepared by inoculation of 10^1^–10^9^ *P. putida* mt‐2 cells per gram soil and subsequently extracting the DNA. Soil without inoculation of mt‐2 cell had a natural background of *xyl* genes; hence, the standard curve was not linear below 10^3^ gene copies per gram of soil. From the slope of the standard curves, the amplification efficiencies of the qPCRs were calculated using the formula *E* = 10^(−1/slope)^−1; for all four gene amplified, the efficiencies were in the range 98–106%. Gene expression was calculated as mRNA normalized per DNA copy numbers as previously described (Nicolaisen *et al*., 2008), taking dilution steps from the DNase‐treatment and RT into consideration when calculating mRNA numbers from the cDNA numbers. DNA and cDNA samples from the soil qPCRs were randomly chosen for Sanger sequencing to verify that the primers only targeted specific products in the soil.

### Statistical analysis

All experiments were repeated independently at least twice, with each independent experiment involving triplicate samples. Mean values of such triplicates from one representative experiment are reported ± standard error of mean. Statistical significance was tested with Student's *t*‐test using the software PAST3.10 (University of Oslo, Norway). Data were considered significant when *P* < 0.05.

## Conflict of interest

The authors have no conflict of interest.

## References

[mbt212404-bib-0001] Aislabie, J.M. , Ryburn, J. , Gutierrez‐Zamora, M.L. , Rhodes, P. , Hunter, D. , Sarmah, A.K. , *et al* (2012) Hexadecane mineralization activity in hydrocarbon‐contaminated soils of Ross Sea region Antarctica may require nutrients and inoculation. Soil Biol Biochem 45: 49–60.

[mbt212404-bib-0002] Aranda‐Olmedo, I. , Ramos, J.L. , and Marqués, S. (2005) Integration of signals through Crc and PtsN in catabolite repression of *Pseudomonas putida* TOL plasmid pWW0. Appl Environ Microbiol 71: 4191–4198. 16085802 10.1128/AEM.71.8.4191-4198.2005 PMC1183334

[mbt212404-bib-0004] Cases, I. , and de Lorenzo, V. (2005) Promoters in the environment: transcriptional regulation in its natural context. Nat Rev Microbiol 3: 105–118. 15685222 10.1038/nrmicro1084

[mbt212404-bib-0005] Cases, I. , Ussery, D.W. , and De Lorenzo, V. (2003) The σ^54^ regulon (sigmulon) of *Pseudomonas putida* . Environ Microbiol 5: 1281–1293. 14641574 10.1111/j.1462-2920.2003.00528.x

[mbt212404-bib-0006] Commichau, F.M. , Forchhammer, K. , and Stülke, J. (2006) Regulatory links between carbon and nitrogen metabolism. Curr Opin Microbiol 9: 167–172. 16458044 10.1016/j.mib.2006.01.001

[mbt212404-bib-0007] Coutts, G. , Thomas, G. , Blakey, D. , and Merrick, M. (2002) Membrane sequestration of the signal transduction protein GlnK by the ammonium transporter AmtB. EMBO J 21: 536–545. 11847102 10.1093/emboj/21.4.536 PMC125854

[mbt212404-bib-0008] Daurabas, D. , and Chakrabarty, A. (1992) The environment, microbes and bioremediation: microbial activities modulated by the environment. Biodegradation 3: 125–135.

[mbt212404-bib-0009] Davis, J.W. , and Madsen, S. (1996) Factors affecting the biodegradation of toluene in soil. Chemosphere 33: 107–130. 8680827 10.1016/0045-6535(96)00152-x

[mbt212404-bib-0010] Del Castillo, T. , and Ramos, J.L. (2007) Simultaneous catabolite repression between glucose and toluene metabolism in *Pseudomonas putida* is channeled through different signaling pathways. J Bacteriol 189: 6602–6610. 17616587 10.1128/JB.00679-07 PMC2045187

[mbt212404-bib-0011] Duetz, W.A. , Marques, S. , Wind, B. , Ramos, J.L. , and van Andel, J.G. (1996) Catabolite repression of the toluene degradation pathway in *Pseudomonas putida* harboring pWWO under various conditions of nutrient limitation in chemostat culture. Appl Environ Microbiol 62: 601–606. 8593060 10.1128/aem.62.2.601-606.1996 PMC167825

[mbt212404-bib-0014] Geisseler, D. , Horwath, W.R. , Joergensen, R.G. , and Ludwig, B. (2010) Pathways of nitrogen utilization by soil microorganisms ‐ A review. Soil Biol Biochem 42: 2058–2067.

[mbt212404-bib-0015] Greated, A. , Lambertsen, L. , Williams, P.A. , and Thomas, C.M. (2002) Complete sequence of the IncP‐9 TOL plasmid pWW0 from *Pseudomonas putida* . Environ Microbiol 4: 856–871. 12534468 10.1046/j.1462-2920.2002.00305.x

[mbt212404-bib-0016] Hendrickx, B. , Junca, H. , Vosahlova, J. , Lindner, A. , Rüegg, I. , Bucheli‐Witschel, M. , *et al* (2006) Alternative primer sets for PCR detection of genotypes involved in bacterial aerobic BTEX degradation: distribution of the genes in BTEX degrading isolates and in subsurface soils of a BTEX contaminated industrial site. J Microbiol Methods 64: 250–265. 15949858 10.1016/j.mimet.2005.04.018

[mbt212404-bib-0017] Hervás, A.B. , Canosa, I. , and Santero, E. (2008) Transcriptome analysis of *Pseudomonas putida* in response to nitrogen availability. J Bacteriol 190: 416–420. 17965157 10.1128/JB.01230-07 PMC2223721

[mbt212404-bib-0018] Hervás, A.B. , Canosa, I. , Little, R. , Dixon, R. , and Santero, E. (2009) NtrC‐dependent regulatory network for nitrogen assimilation in *Pseudomonas putida* . J Bacteriol 191: 6123–6135. 19648236 10.1128/JB.00744-09 PMC2747892

[mbt212404-bib-0019] Hervás, A.B. , Canosa, I. , and Santero, E. (2010) Regulation of glutamate dehydrogenase expression in *Pseudomonas putida* results from its direct repression by NtrC under nitrogen‐limiting conditions. Mol Microbiol 78: 305–319. 20735780 10.1111/j.1365-2958.2010.07329.x

[mbt212404-bib-0020] Huang, W.E. , Singer, A.C. , Spiers, A.J. , Preston, G.M. , and Whiteley, A.S. (2008) Characterizing the regulation of the Pu promoter in *Acinetobacter baylyi* ADP1. Environ Microbiol 10: 1668–1680. 18363715 10.1111/j.1462-2920.2008.01583.x

[mbt212404-bib-0021] Javelle, A. , and Merrick, M. (2005) Complex formation between AmtB and GlnK: an ancestral role in prokaryotic nitrogen control. Biochem Soc Trans 33: 170–172. 15667297 10.1042/BST0330170

[mbt212404-bib-0022] Jensen, L.E. , and Nybroe, O. (1999) Nitrogen availability to *Pseudomonas fluorescens* DF57 is limited during decomposition of barley straw in bulk soil and in the barley rhizosphere. Appl Environ Microbiol 65: 4320–4328. 10508054 10.1128/aem.65.10.4320-4328.1999 PMC91572

[mbt212404-bib-0024] Koch, B. , Worm, J. , Jensen, L.E. , Hojberg, O. , and Nybroe, O. (2001) Carbon limitation induces σ^S^‐dependent gene expression in *Pseudomonas fluorescens* in soil. Appl Environ Microbiol 67: 3363–3370. 11472905 10.1128/AEM.67.8.3363-3370.2001 PMC93029

[mbt212404-bib-0026] Komilis, D.P. , Vrohidou, A.E.K. , and Voudrias, E.A. (2010) Kinetics of aerobic bioremediation of a diesel‐contaminated sandy soil: effect of nitrogen addition. Water Air Soil Pollut 208: 193–208.

[mbt212404-bib-0027] Leigh, J.A. , and Dodsworth, J.A. (2007) Nitrogen regulation in bacteria and archaea. Annu Rev Microbiol 61: 349–377. 17506680 10.1146/annurev.micro.61.080706.093409

[mbt212404-bib-0028] Lindstrom, J.E. , Prince, R.C. , Clark, J.C. , Grossman, M.J. , Yeager, T.R. , Braddock, J.F. , and Brown, E.J. (1991) Microbial populations and hydrocarbon biodegradation potentials in fertilized shoreline sediments affected by the T/V Exxon Valdez oil spill. Appl Environ Microbiol 57: 2514–2522. 1662935 10.1128/aem.57.9.2514-2522.1991 PMC183612

[mbt212404-bib-0029] de Lorenzo, V. (2008) Systems biology approaches to bioremediation. Curr Opin Biotechnol 19: 579–589. 19000761 10.1016/j.copbio.2008.10.004

[mbt212404-bib-0030] de Lorenzo, V. , Pieper, D. , and Ramos, J.L. (2013) From the test tube to the environment ‐ and back. Environ Microbiol 15: 6–11. 23286645 10.1111/j.1462-2920.2012.02896.x

[mbt212404-bib-0031] Magasanik, B. (1993) The regulation of nitrogen utilization in enteric bacteria. J Cell Biochem 51: 34–40. 8094391 10.1002/jcb.240510108

[mbt212404-bib-0032] Martínez‐Lavanchy, P.M. , Müller, C. , Nijenhuis, I. , Kappelmeyer, U. , Buffing, M. , McPherson, K. , and Heipieper, H.J. (2010) High stability and fast recovery of expression of the TOL plasmid‐carried toluene catabolism genes of *Pseudomonas putida* mt‐2 under conditions of oxygen limitation and oscillation. Appl Environ Microbiol 76: 6715–6723. 20709833 10.1128/AEM.01039-10 PMC2953008

[mbt212404-bib-0033] Meckenstock, R.U. , Elsner, M. , Griebler, C. , Lueders, T. , Stumpp, C. , Dejonghe, W. , *et al* (2015) Biodegradation: updating the concepts of control for microbial clean‐up in contaminated aquifers. Environ Sci Technol 49: 7073–7081. 26000605 10.1021/acs.est.5b00715

[mbt212404-bib-0034] Merrick, M.J. , and Edwards, R.A. (1995) Nitrogen control in bacteria. Microbiol Rev 59: 604–622. 8531888 10.1128/mr.59.4.604-622.1995 PMC239390

[mbt212404-bib-0035] Moreno‐Vivián, C. , Cabello, P. , Blasco, R. , and Castillo, F. (1999) Prokaryotic nitrate reduction : molecular properties and functional distinction among bacterial nitrate reductases. J Bacteriol 181: 6573–6584. 10542156 10.1128/jb.181.21.6573-6584.1999 PMC94119

[mbt212404-bib-0036] Nicolaisen, M.H. , Bælum, J. , Jacobsen, C.S. , and Sørensen, J. (2008) Transcription dynamics of the functional *tfdA* gene during MCPA herbicide degradation by *Cupriavidus necator* AEO106 (pRO101) in agricultural soil. Environ Microbiol 10: 571–579. 18190516 10.1111/j.1462-2920.2007.01476.x

[mbt212404-bib-0037] Pflüger‐Grau, K. , and Görke, B. (2010) Regulatory roles of the bacterial nitrogen‐related phosphotransferase system. Trends Microbiol 18: 205–214. 20202847 10.1016/j.tim.2010.02.003

[mbt212404-bib-0038] Poblete‐Castro, I. , Escapa, I.F. , Jäger, C. , Puchalka, J. , Chi Lam, C. , Schomburg, D. , *et al* (2012) The metabolic response of *P. putida* KT2442 producing high levels of polyhydroxyalkanoate under single‐ and multiple‐nutrient‐limited growth: highlights from a multi‐level omics approach. Microb Cell Fact 11: 34. 22433058 10.1186/1475-2859-11-34 PMC3325844

[mbt212404-bib-0039] Ramos, J.L. , Marqués, S. , and Timmis, K.N. (1997) Transcriptional control of the *Pseudomonas* TOL plasmid catabolic operons is achieved through an interplay of host factors and plasmid‐encoded regulators. Annu Rev Microbiol 51: 341–373. 9343354 10.1146/annurev.micro.51.1.341

[mbt212404-bib-0040] Rocca, J.D. , Hall, E.K. , Lennon, J.T. , Evans, S.E. , Waldrop, M.P. , Cotner, J.B. , *et al* (2014) Relationships between protein‐encoding gene abundance and corresponding process are commonly assumed yet rarely observed. ISME J 9: 1693–1699. 25535936 10.1038/ismej.2014.252 PMC4511926

[mbt212404-bib-0041] Shingler, V. (2003) Integrated regulation in response to aromatic compounds: from signal sensing to attractive behaviour. Environ Microbiol 5: 1226–1241. 14641570 10.1111/j.1462-2920.2003.00472.x

[mbt212404-bib-0042] Svenningsen, N.B. , Pérez‐Pantoja, D. , Nikel, P.I. , Nicolaisen, M.H. , de Lorenzo, V. , and Nybroe, O. (2015) *Pseudomonas putida* mt‐2 tolerates reactive oxygen species generated during matric stress by inducing a major oxidative defense response. BMC Microbiol 15: 202. 26445482 10.1186/s12866-015-0542-1 PMC4595014

[mbt212404-bib-0043] van Veen, J.A. , van Overbeek, L.S. , and van Elsas, J.D. (1997) Fate and activity of microorganisms introduced into soil. Microbiol Mol Biol Rev 61: 121–135. 9184007 10.1128/mmbr.61.2.121-135.1997 PMC232604

[mbt212404-bib-0044] Velázquez, F. , de Lorenzo, V. , and Valls, M. (2006) The *m*‐xylene biodegradation capacity of *Pseudomonas putida* mt‐2 is submitted to adaptation to abiotic stresses: evidence from expression profiling of *xyl* genes. Environ Microbiol 8: 591–602. 16584471 10.1111/j.1462-2920.2005.00936.x

[mbt212404-bib-0045] Walecka‐Hutchison, C.M. , and Walworth, J.L. (2006) Assessment of C: N ratios and water potential for nitrogen optimization in diesel bioremediation. Bioremediat J 10: 25–35.

[mbt212404-bib-0047] Yeom, S. , Yeom, J. , and Park, W. (2010) NtrC‐sensed nitrogen availability is important for oxidative stress defense in *Pseudomonas putida* KT2440. J Microbiol 48: 153–159. 20437145 10.1007/s12275-010-0075-0

